# Reversals in initially denied Department of Veterans Affairs’ PTSD disability claims after 17 years: a cohort study of gender differences

**DOI:** 10.1186/s12905-021-01214-7

**Published:** 2021-02-16

**Authors:** Maureen Murdoch, Michele Roxanne Spoont, Nina Aileen Sayer, Shannon Marie Kehle-Forbes, Siamak Noorbaloochi

**Affiliations:** 1 Section of General Internal Medicine, Minneapolis VA Health Care System, One Veterans Drive (111-0), Minneapolis, MN 55417 USA; 2 Center for Care Delivery and Outcomes Research, Minneapolis VA Health Care System, One Veterans Drive (152), Minneapolis, MN 55417 USA; 3 Department of Internal Medicine, University of Minnesota Medical School, 420 Delaware St SE, Minneapolis, MN 55455 USA; 4 National Centers for PTSD, Pacific Islands Division, Department of Veterans Affairs, 3375 Koapaka Street, Suite I-560, Honolulu, HI 96819 USA; 5 Department of Psychiatry, University of Minnesota Medical School, F282/2A West Building, 2450 Riverside Avenue S, Minneapolis, MN 55454 USA; 6 National Center for PTSD Women’s Health Sciences Division at VA Boston Healthcare System, 150 S Huntington Ave, Boston, MA 02130 USA

**Keywords:** Posttraumatic stress disorder, Gender, Cohort studies, Veterans disability claims, Compensation

## Abstract

**Background:**

In 2011, the Department of Veterans Affairs (VA) strengthened its disability claims processes for military sexual trauma, hoping to reduce gender differences in initial posttraumatic stress disorder (PTSD) disability awards. These process improvements should also have helped women reverse previously denied claims and, potentially, diminished gender discrepancies in appealed claims’ outcomes. Our objectives were to examine gender differences in reversals of denied PTSD claims’ outcomes after 2011, determine whether disability awards (also known as “service connection”) for other disorders offset any PTSD gender discrepancy, and identify mediating confounders that could explain any persisting discrepancy.

**Methods:**

From a nationally representative cohort created in 1998, we examined service connection outcomes in 253 men and 663 women whose initial PTSD claims were denied. The primary outcome was PTSD service connection as of August 24, 2016. Secondary outcomes were service connection for any disorder and total disability rating. The total disability rating determines the generosity of Veterans’ benefits.

**Results:**

51.4% of men and 31.3% of women were service connected for PTSD by study’s end (*p* < 0.001). At inception, 54.2% of men and 63.2% of women had any service connection—i.e., service connection for disorders other than PTSD (*p* = 0.01) and similar total disability ratings (*p* = 0.50). However, by study’s end, more men than women had any service connection (88.5% versus 83.5%, *p* = 0.05), and men’s mean total disability rating was substantially greater than women’s (77.1 ± 26.2 versus 66.8 ± 30.7, *p* < 0.001). History of military sexual assault had the largest effect modification on men’s versus women’s odds of PTSD service connection.

**Conclusion:**

Even after 2011, cohort men were more likely than the women to reverse initially denied PTSD claims, and military sexual assault history accounted for much of this difference. Service connection for other disorders initially offset women’s lower rate of PTSD service connection, but, ultimately, men’s total disability ratings exceeded women’s. Gender discrepancies in service connection should be monitored beyond the initial claims period.

**Supplementary Information:**

The online version contains supplementary material available at 10.1186/s12905-021-01214-7.

## Background

The Department of Veterans Affairs’ (VA) Veterans Benefits Administration (VBA) directs the United States’ third largest federal disability program, second only to the Social Security Disability Insurance and Supplemental Security Income programs. Veterans indemnified by VA for disorders incurred or aggravated by military service receive disability ratings ranging from 0% (non-disabled) to 100% (completely disabled). Higher ratings are associated with greater compensation and other benefits. Veterans may be indemnified (“service connected” in VA parlance) for multiple disorders, but their total disability rating can never exceed 100%. The total disability rating is also called the “combined degree of service connection.” Veterans denied service connection have multiple avenues to appeal their rulings. They may also open new claims for the same condition if they are able to provide new and material evidence.

Of the 4.4 million Veterans service-connected for military-related disorders in 2016, almost 20% were indemnified for posttraumatic stress disorder (PTSD) [1]. Compared to Veterans who apply for but do not receive PTSD service connection, successful claimants are more likely to engage with outpatient VA mental health services, to demonstrate clinically relevant improvements in PTSD symptoms 10 years later, and to have less poverty and homelessness [2–5]. PTSD service connection is especially important in reducing poverty for men who self-identify as disabled or as African American [6]. PTSD service connection therefore influences well-being across multiple domains, and it is critical that all Veterans eligible for these benefits receive them.

Historically, 71% of women filing VA PTSD disability claims do so based on military sexual assault experiences, whereas more than 90% of men file PTSD disability claims based on combat exposure [7]. As a result, women are granted PTSD service connection less frequently than men are [7, 8]: it is simply easier to establish evidence of combat exposure than of military sexual assault [9, 10]. Although VBA had developed strategies to counter this difficulty since the early 1990s [11], in 2011 they distributed further guidance and training to the field to “ensure consistency and fairness” in developing military sexual trauma claims [12, p. 3]. Specifically, when witnesses and police reports did not exist, claims processors were charged to look for alternative markers of sexual assault, such as sudden, proximate behavior changes (e.g., upticks in drinking, taking more sick leave), requests for job transfers, or requests for HIV or pregnancy testing, to name just a few. Aimed at initial claims, this development could also have encouraged more women with previously denied PTSD disability claims to file appeals and, once filed, to prevail.

Our first goal was to examine the impact of this 2011 initiative on previously denied claims by taking advantage of a long-standing, well-characterized cohort of men and women who applied for PTSD disability benefits between 1994 and 1998 [4, 7, 13]. As of 2007, 50% of men in this cohort, compared to 42% of women, had successfully reversed their initial PTSD claims’ denials [14]. Here, we re-visit the cohort to see if men and women’s reversal rates would be more similar after 2011.

Our second goal was to see if service connection for other disorders might offset any persisting gender differences. In 2010, the VA’s Office of the Inspector General [8], found that, at the time of initial claim, women were less likely than men to receive PTSD service connection but more likely to receive service connection for other mental disorders. They argued that this substitution prevented women from experiencing a net welfare loss. If men filing PTSD disability claims received 70% disability ratings for PTSD, for example, while women filing PTSD disability claims received 70% disability ratings for depression, then their base compensation packages would be the same. Such substitutions could explain why, 2 years after filing an initial PTSD disability claims, poverty rates were similar in women with and without PTSD service connection [6]. Because the present study focuses exclusively on Veterans with denied initial claims, we anticipated, based on the 2010 VA Inspector General report, that the women would be more likely than the men to be service connected for other disorders. For net parity, however, men and women’s total disability rating would need to be similar.

Our third goal was to identify reasons for any persistent gender discrepancies. We have previously shown in this cohort that older age, white race, service during the Vietnam Conflict, married status, and having a VA chart diagnosis of PTSD were each associated with higher odds of obtaining initial PTSD service connection or with successfully reversing a denial pre-2011 [14–16]. It made sense that these effects would carry through after 2011. A history of combat exposure instead of military sexual assault also predicted lower odds of PTSD service connection upon initial claim [7] but was less influential on odds of successfully reversing a denied claim prior to 2011 [14]. In the present analysis, our goal was to determine whether trauma type affected claims decisions at all after the 2011 guidance.

## Methods

### Study design

The study is a post hoc, secondary analysis of an ambispective cohort (ambispective = retrospective and prospective elements).

### Participants

Between 1998 and 2000, we randomly selected 2700 men and 2700 women (total *N* = 5400) from a population of 100,750 men and 3866 women who applied for Department of Veterans Affairs PTSD disability benefits between 1994 and 1998. The inception cohort are the 1654 men and 1683 women who responded to a mailed survey between 1998 and 2000. We assessed service connection outcomes for the cohort on Dec 31, 1999 (*Time 1*), April 30, 2007 (*Time 2*), and August 24, 2016 (*Time 3*). The present study is limited to the 253 men and 663 women cohort members whose original, Time 1, PTSD disability claims were denied and who were alive at Time 3. Women outnumber the men in the present study because they were substantially more likely to have their initial PTSD disability claims denied. The Minneapolis VA Health Care System’s Human Studies Subcommittee reviewed and approved the study protocol.

### Outcomes

The primary outcome was Veterans’ PTSD service-connected status as of August 24, 2016 (Time 3). Secondary outcomes included Veterans’ service-connected status for any disorder at all three time points, and their total disability rating (combined degree of service connection) at all 3 time points. Note that, for Veterans who reversed their PTSD claims’ denials, the Time 2 and 3 total disability rating will include their service rating for PTSD as well as for other disorders.

### Main independent variable

The independent variable of interest was gender, dichotomized as male or female. Gender was obtained at Time 1 from the Veterans Issues Tracking Adjudication Log.

### Potential confounders

Confounders available for the entire study sample included: age, race, service era, combat and military sexual trauma exposures, married status at Time 1, and having at least one VA chart diagnosis of PTSD between 1994 and 2006.

### Data sources

We used VA administrative databases to obtain Veterans’ age, service era (categorized as during the Vietnam Conflict versus later), and service connection information. To determine if Veterans had been diagnosed with PTSD between 1994 and 2006, we used International Classification of Diseases, Ninth Revision, Clinical Modification (ICD-CM-9) codes housed in the VA National Patient Care Database. Veterans with any ICD-CM-9 code for PTSD between 1994 and 2006 were categorized as having a chart diagnosis of PTSD.

We used self-reported, single-item questions from the Time 1 survey to determine Veterans’ married status and race. The Time 1 survey also assessed Veterans’ combat exposure using a modified, 22-item version of the Combat Exposure Index [17] and military sexual assault history, using a modified, 4-item version of the “criminal sexual misconduct” subscale from the Sexual Harassment Inventory [18]. To be consistent with our prior analyses, combat and military sexual assault were dichotomized into “any” versus “none” variables.

### Analysis

We used χ^2^ tests and ANOVA to compare unadjusted gender differences in the primary and secondary outcomes. To identify potential confounders, we first used χ^2^ tests and ANOVA to compare characteristics between those who were and were not service connected for PTSD at Time 3 and to compare men and women’s characteristics. We then used sufficient summary analysis [19, 20] to assess the direct effect of gender on Veterans’ odds of Time 3 PTSD service connection after controlling for identified confounders. To this end, we used sufficient summary score analysis both as subclassification on dimension-reducing, sufficient summary scores and as a covariance adjustment.

Sufficient summary scores are an extension of propensity analysis [21]. To develop dimension-reducing, sufficient summary scores, all predictors associated with Time 3 PTSD service connection in unadjusted analyses at p ≤ 0.10 were simultaneously submitted to multiple logistic regression within each gender (see Additional file 1: Tables S1 and S2). Age correlated 0.81 with service before or after the Vietnam Conflict; therefore, only service before or after the Vietnam Conflict was included in analyses. Correlations among the other predictors ranged from − 0.35 to 0.24. Variables associated with Time 3 PTSD service connection at p ≤ 0.05 in either gender were retained such that the final four predictors used to create sufficient summary scores and to assess confounding were: service during or after the Vietnam Conflict, chart diagnosis of PTSD, history of military sexual assault, and history of combat. Thus, each Veteran’s summary score corresponded to his or her probability of Time 3 PTSD service connection, based on their unique combination of the 4 variables. For the subclassification analysis, we created 3 summary score strata instead of the usual 5 because of sparse data. The summary score strata ranged from low probability (< 0.38, *n* = 347) of PTSD service connection to medium probability (≥ 0.38 and ≤ 0.55, *n* = 341) to high probability (> 0.55, *n* = 228).

We also examined the degree to which each of the four variables changed the unadjusted association between gender and Time 3 PTSD service connection. If the gender effect size changed 10% or more after adjustment, we considered that evidence of mediating confounding [22]. The change in effect size was calculated as the absolute difference between the unadjusted and adjusted association between gender and Time 3 PTSD service connection, divided by the unadjusted association and multiplied by 100%. We also report the study’s E-value [23] with 95% confidence intervals (CI). E-value measures how high an association between unmeasured mediating confounder(s), gender, and PTSD service connection would need to be to explain away any remaining associations between gender and Time 3 PTSD service connection after adjusting for the sufficient summary score. We used SPSS Version 19 for all analyses.

## Results

### Sample descriptives

Men’s mean age when they originally filed their claims was 45.5 (± 7.2) years, and women’s, 37.2 (± 8.5) years. By Time 3, men’s mean age was 65.3 (± 7.2) years and women’s, 57.1 (± 8.6) years (*p*s < 0.001). Table 1 shows the sample’s categorical sociodemographic characteristics, stratified by those who did and did not become service connected for PTSD by Time 3 and stratified by gender. As seen from Table 1, relative to their counterparts, Veterans who became service connected for PTSD by Time 3 were more likely to have served during the Vietnam Conflict, to have a combat history, and to have a PTSD chart diagnosis. Many of these predictors, particularly service during Vietnam, combat exposure, and military sexual assault history, also differed by gender.

**Table 1:** Sociodemographic Characteristics of the Sample, Stratified by Time 3 PTSD Service Connection Status and Stratified by Sex

Characteristic	Time 3 PTSD service connection status		Sex	
	SC − N = 579	SC + N = 337	Men N = 253	Women N = 663
	*n* (%)	*n* (%)	*n* (%)	*n* (%)
*Race/ethnicity*				
White	371 (64.1%)	220 (65.3%)***	164 (64.8%)	427 (64.4%)***
Black or African American	150 (25.9%)	87 (25.8%)***	64 (25.3%)	173 (26.1%)***
Hispanic	31 (5.4%)	17 (5.0%)***	16 (6.3%)	32 0(4.8%)***
Other	46 (7.9%)	31 (9.2%)***	19) 0(7.5%	58 0(8.7%)***
Served during Vietnam Conflict	199 (34.4%)	167 (49.6%)***	207 (81.8%)	159 (24.0%)***
Married at Time 1	230 (60.3%)	124 (36.8%)***	122 (48.2%)	232 (35.0%)***
History of combat exposure	220 (38.0%)	184 (54.6%)***	220 (87.0%)	184 (27.8%)***
History of military sexual assault	273 (47.2%)	173 (51.3%)***	16 (6.3%)	430 (64.9%)***
Any PTSD diagnosis, 1994–2006	328 (56.6%)	301 (89.3%)***	180 (71.1%)	449 (67.7%)***

*PTSD* posttraumatic stress disorder, *SC − *not service connected, *SC* + is service connected

*p < 0.05; **p < 0.01; ***p < 0.001

### Changes in PTSD service connection

**Fig. 1:**
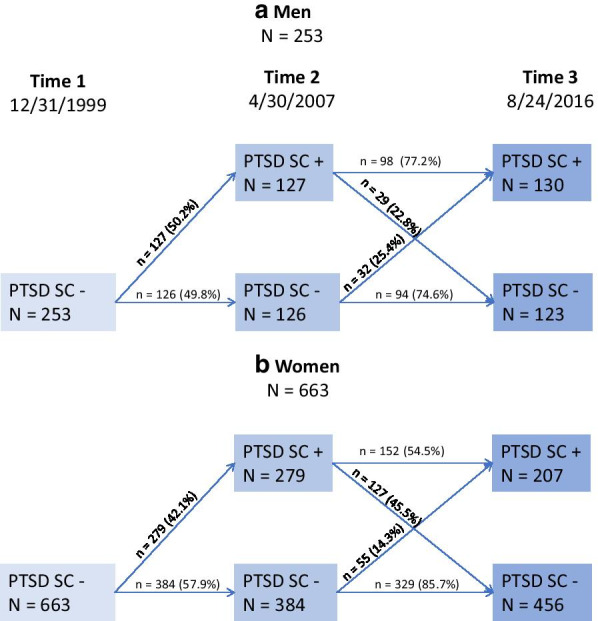
Change in PTSD service connection over time. *PTSD* posttraumatic stress disorder, *SC* + is service connected for PTSD, *SC − *is not service connected for PTSD

Figure 1 shows that PTSD service connection status was dynamic, with individual gains and losses over the study’s course. Of the 127 men and 279 women who gained PTSD service connection at Time 2, women were twice as likely as the men to have lost it again by Time 3. The net gain in PTSD service connection by Time 3 was 130 (51.4%) men and 207 (31.3%) women.

### Other service connection outcomes

Although women were less likely to be service connected for PTSD at any time point, Table 2 shows that, as expected, statistically significantly more women than men were service connected for disorders other than PTSD at Time 1; their mean total disability rating (combined degree of service connection) was about the same as men’s. Over time, however, men’s net total disability rating increased at a higher rate than did the women’s and was substantially higher than the women’s by Time 3. Additional file 1: Figure S1 shows that, for both men and women, total disability ratings skewed rightward with time. However, a greater proportion of men than women had total disability ratings of 100% by Time 3.

**Table 2:** Service connection outcomes over time

Service connection outcomes	Men N = 253	Women N = 663	p value
	*n (%)*	*n (%)*	
*Service connected for any disorder*			
At Time 1^a^	137 (54.2)	419 (63.2)	0.01
At Time 2	201 (79.4)	506 (76.3)	0.05
At Time 3	224 (88.5)	553 (83.5)	0.05

	Mean (SD)	Mean (SD)	
*Total disability ratings (combined degree of service connection)*^*b*^			
At Time 1	25.4 (27.4)	27.0 (23.3)	0.50
At Time 2	65.3 (28.4)	57.8 (28.9)	0.001
At Time 3	77.1 (26.2)	66.8 (30.7)	< 0.001

^a^No one in the sample was service connected for posttraumatic stress disorder at Time 1

^b^Among those with any service connection for any disorder

### Sufficient summary analysis and confounding

Overall, men’s mean sufficient summary score, which corresponds to their mean probability of Time 3 PTSD service connection, was 0.45 ± 0.19, compared to women’s mean sufficient summary score of 0.34 ± 0.18 (*p* < 0.001). Stratifying by sufficient summary class improved balance in observed variables between those who were and were not service connected for PTSD at Time 3 (Additional file 1: Table S3), but imbalances in the highest stratum remained. The association, β, between gender and Time 3 PTSD service connection was 0.46, with mean weighted variance = 0.04; *z* = 2.42, *p* = 0.02. This analysis therefore suggested that men had statistically significantly higher odds of Time 3 PTSD service connection than did women after controlling for available confounders.

Table 3 shows the modifying effect of each potential confounder on gender’s association with Time 3 PTSD service connection. The final row shows results of covariance adjustment on the sufficient summary score, which indicated that men had 1.52 higher odds of gaining PTSD service connection at Time 3 than did women after controlling for all available confounders. Only chart diagnosis of PTSD failed to meet criterion for confounding. History of military sexual assault had the largest impact on the odds ratio between male gender and PTSD service connection. When military sexual assault was included in the model, the odds of men being service connected for PTSD compared to women increased from an unadjusted odds ratio of 2.33 to an adjusted odds ratio of 4.14 (77.7% effect modification). Adjusting for all the predictors simultaneously using the sufficient summary score resulted in an overall, 34.8% effect modification on the association between gender and PTSD service connection. E-value for the association between gender and Time 3 PTSD service connection was 1.77 (95% CI 1.28).

**Table 3:** Sex’s association with time 3 PTSD service connection after controlling for other potential confounders

Controlling for:	Association between gender and PTSD service connection							Size of effect modification (%)
	β	SE	Wald χ^2^	df	p value	OR	95% CI	
Nothing (unadjusted)	0.845	0.15	31.26	1	< 0.001	2.33	1.73, 3.13	–
Service during Vietnam	0.672	0.18	14.32	1	< 0.001	1.96	1.38, 2.77	15.9
PTSD chart diagnosis	0.907	0.16	30.81	1	< 0.001	2.48	1.80, 3.41	6.4
Military sexual assault history	1.420	0.20	51.97	1	< 0.001	4.14	2.81, 6.09	77.7
Combat history	0.630	0.18	12.55	1	< 0.001	1.88	1.33, 2.66	19.3
Sufficient summary score^a^	0.420	0.17	6.284	1	0.01	1.52	1.10, 2.11	34.8

*PTSD* posttraumatic stress disorder, *SE* standard error, *OR* odds ratio; OR > 1 indicates men have greater odds of having PTSD service connection at Time 3 than women. *CI* confidence intervals

– Not applicable

^a^Sufficient summary score simultaneously adjusts for service during Vietnam, PTSD chart diagnosis, military sexual assault history, and combat history

## Discussion

Although substantial proportions of men and women in this cohort ultimately became service connected for PTSD after 2011, the men were significantly more likely to do so than women. Consistent with the VA’s Office of the Inspector General’s [8] 2010 report, men and women had the same net, total disability rating at Time 1. From Time 2 forward, however, men’s net total disability ratings persistently exceeded women’s. Therefore, service ratings for other disorders did not offset women’s lower, long-term odds of PTSD service connection.

In the present study, three predictors—service during Vietnam, history of military sexual assault, and history of combat—were unevenly distributed by gender and explained some, but not all, of the gender difference in Time 3 PTSD service connection. Chart diagnosis of PTSD independently predicted Time 3 PTSD service connection but was evenly distributed by gender. History of military sexual assault had the largest confounding effect on the association between gender and Time 3 PTSD service connection, followed by combat exposure. This is perhaps unsurprising, because, by law, Veterans’ self-report is enough to establish credible evidence of combat exposure if it is “consistent with the circumstances, conditions, or hardships of [their] service” [9], but self-report is not enough to establish credible evidence of military sexual assault [10]. As women are more likely than men to file military sexual assault claims, much of the gender discrepancy in initial claims’ approvals has been attributed to this disparate burden of proof [8]. Despite VBA’s attempt to reduce the challenges associated with military sexual assault claims, our data indicates that gender discrepancies carried into the appeals process, even after 2011. More recently, the Office of Inspector General [12] identified processing errors in almost half the military sexual trauma claims denied in fiscal year 2017. Whether these errors might have applied to even earlier years—thus contributing to our observed gender differences, is uncertain. We must also acknowledge that, had the VBA not implemented stronger processes for military sexual assault claims, the gender differences seen here may have been even greater.

### Strengths and limitations

To our knowledge, this is the longest-running study of PTSD service connection outcomes available. Our reliance on administrative and inception survey data to define confounders was a strength in that we had no missingness in those variables. Each candidate confounder had varied by gender in previous work and was either known or hypothesized to be associated with PTSD service connection. Nonetheless, it seems likely that we had an insufficient set of confounders to fully explain observed gender differences in Time 3 PTSD service connection. One might easily imagine some set of unmeasured variables associated with both gender and Time 3 PTSD service connection at an odds ratio of 1.77. In an earlier analysis, milder PTSD symptoms and better net functioning partially explained why women were less likely than the men to have Time 2 PTSD service connection [14]. We did not have access to similar variables for the entire cohort at Time 3. Thus, the observed, persistent gender difference in service connection outcomes at Time 3 could be at least partially attributable to gender differences in clinical recovery.

In one regional cohort comprised of 99% men, 83% of Veterans denied service connection for PTSD said they planned to appeal [2]. However, we do not know if these Veterans’ intentions generalize nationally or to women or if Veterans followed through with their stated intentions to appeal. Relative to men, women may be less likely to file claims’ appeals or to access skilled advocates during the appeals process. If so, these could be potential intervention points for narrowing the gender gap we observed here. Furthermore, to our knowledge, VBA did not widely advertise their 2011 process changes. Women with claims denied prior to 2011 may not have known about the changes and thus may not have filed subsequent appeals. This study did not have access to Veterans’ claims appeals data. Since appeals play a causal role in reversing denied disability claims, future studies should specifically test for gender differences in appeal rates and outcomes.

We created this cohort based on the calendar years in which Veterans initially applied for PTSD service connection. The sample is representative of claimants from those years, but cohort men and women nonetheless differed substantially on many characteristics, complicating direct comparisons. Despite a reasonably large sample, sufficient summary analysis could not overcome problems with sparse data in our highest stratum of probability for PTSD service connection. Selecting men and women from the same service era might improve their comparability and reduce sparse data problems in future research. The present cohort excludes post-9/11 Veterans: findings may not apply to them.

### Policy implications

Women are now the fastest growing demographic of US Veterans and account for almost 15% of active duty troops [24]. When the affected population is large, as in this case, even small disparities may carry large impacts. We have shown that, even after 17 years, the appeals or reapplication process does not reduce initial gender differences in PTSD service connection. However, women Veterans with previously denied claims may not have been made aware of the 2011 changes in VBA’s procedures. For these reasons, attention to claims outcomes should not be limited to just initial claims. While service connection for other disorders may have offset gender differences in PTSD service connection at first, men’s combined degree of service connection eventually exceeded women’s. Because PTSD service connection is associated with several important outcomes, including treatment engagement and symptom reduction [2–5, 25], we must ensure that observed gender differences reflect appropriate sources of variation. Besides service during Vietnam, military sexual assault history, and combat exposure, there are probably multiple other variables that differ by gender and influence long-term PTSD service connection. Identifying and addressing them would ensure equity, transparency, and confidence in the VA’s indemnity program.

## Conclusions

Planned, prospective investigations are needed to understand how Veterans’ service-connected status changes over time—for PTSD and for other disorders—and how they differ by gender. These studies should include in-depth assessments of Veterans’ clinical characteristics, such as disease severity; process variables, such as how initial claims, re-evaluations and appeals are handled; and associated outcomes, such as homelessness and work, role, and social functioning.

## Supplementary Information

**Additional file 1**: **Table S1**. Characteristics associated with time 3 PTSD service connection within each sex. **Table S2**. Predictors of Time 3 PTSD service connection for men and for women. Results of multiple logistic regression. **Table S3**. Predictors of Time 3 PTSD Service connection after stratifying by sufficient summary class. **Figure S1**. Change in the distribution of total disability ratings by time, stratified by sex.

## Data Availability

The data that support the findings of this study are available from the corresponding author (MM) upon reasonable request and with local Minneapolis VA Health Care System IRB permission.

## Abbreviations

ANOVA: Analysis of variance
CI: Confidence intervals
ICD-9-CM: International Classification of Diseases, Clinical Modification, 9th revision
PTSD: Posttraumatic stress disorder
SC +: Is service connected for PTSD
SC −: Is not service connected for PTSD
VA: Department of Veterans Affairs
VBA: Veterans Benefits Administration
