# Supplementary material for: Reversals in initially denied Department of Veterans Affairs’ PTSD disability claims after 17 years: a cohort study of gender differences
Source: BMC Womens Health. 2021 Feb 16;21:70. doi: 10.1186/s12905-021-01214-7 (PMC7885341; doi:10.1186/s12905-021-01214-7)
Supplement: Supplementary file 1 — Additional file 1: Table S1. Characteristics associated with time 3 PTSD service connection within each sex. Table S2. Predictors of Time 3 PTSD service connection for men and for women. Results of multiple logistic regression. Table S3. Predictors of Time 3 PTSD Service connection after stratifying by sufficient summary class. Figure S1. Change in the distribution of total disability ratings by time, stratified by sex. [file 12905_2021_1214_MOESM1_ESM.docx]

**Supplementary Table 1.**

Characteristics associated with Time 3 PTSD Service connection within each Sex

| Characteristic | Men  N = 253 | | | Women  N = 663 | | |
| --- | --- | --- | --- | --- | --- | --- |
|  | Time 3 PTSD Service Connection Status | | *p*-value | Time 3 PTSD Service Connection Status | | *p*-value |
|  | SC-  *n* = 123 | SC+  *n* = 130 |  | SC-  *n* = 456 | SC+  *n* = 207 |  |
| Time 1 age < 55 years | 95.9 | 97.7 | 0.43 | 98.7 | 99.0 | 0.70 |
| Race/Ethnicity |  |  |  |  |  |  |
| White | 63.4 | 66.2 | 0.650 | 64.3 | 64.7 | 0.910 |
| Black or African American | 24.4 | 26.2 | 0.750 | 26.3 | 25.6 | 0.850 |
| Hispanic | 7.3 | 5.4 | 0.530 | 4.8 | 4.8 | 1.000 |
| Other | 7.3 | 7.7 | 0.910 | 8.1 | 10.1 | 0.390 |
| Served during Vietnam Conflict | 77.2 | 86.2 | 0.070 | 22.8 | 26.6 | 0.290 |
| Married at inception | 48.0 | 48.5 | 0.940 | 37.5 | 29.5 | 0.050 |
| Combat | 83.7 | 90.0 | 0.14 | 25.7 | 32.4 | 0.07 |
| Sexual assault | 1.6 | 10.8 | 0.003 | 59.4 | 76.8 | <0.001 |
| Any PTSD diagnosis, 1994-2006 | 51.2 | 90.0 | <0.001 | 58.1 | 88.9 | <0.001 |

Results are reported as column percentages (%). PTSD = posttraumatic stress disorder. SC- = not service connected

for PTSD; SC+ = is service connected for PTSD. *p* tests for statistically significant differences between SC+ and SC-

Veterans within sex.

**Supplementary Table 2.**

Predictors of Time 3 PTSD Service Connection for Men and for Women. Results of Multiple

Logistic Regression

| Predictor | Men  N = 253 | | Women  N = 663 | |
| --- | --- | --- | --- | --- |
|  | AOR (95% CI) | p-value | AOR (95% CI) | p-value |
| Served during the Vietnam Conflict | 2.33 (1.09, 4.96) | 0.03 | -- | -- |
| Married at Time 1 | -- | -- | 0  0.77 (0.53, 1.13) | 0.180 |
| Military Sexual Assault History | 10.54 (2.00, 55.50) | 0.005 | 1.78 (1.20, 2.65) | 0.005 |
| Combat history | -- | -- | 1.46 (0.99, 2.14) | 0.054 |
| PTSD dx, 1994-2006 | 9.01 (4.46, 18.18) | <0.001 | 5.02 (3.10, 8.08) | <0.001 |

AOR = Adjusted Odds Ratio for being service connected for posttraumatic stress disorder after

adjusting for the other predictors in the model. AOR > 1 indicates higher odds of being service

connected if one has the predictor relative to one who does not have the predictor.

CI = Confidence Intervals. PTSD = posttraumatic stress disorder. --- Not in model for that gender.

Men’s Hosmer-Lemeshow χ^2^ was 1.87 df = 4 p = 0.76; women’s Hosmer-Lemeshow χ^2^ was 12.34; df = 6; p = 0.06.

**Supplementary Table 3.**

Predictors of Time 3 PTSD Service Connection after Stratifying by Sufficient Summary Class

| Predictor | Time 3 PTSD Service Connection Status | | p-value |
| --- | --- | --- | --- |
|  | SC-  N = 579 | SC+  N = 337 |  |
|  | *n* (%) | *n* (%) |  |
| Served during Vietnam Conflict |  |  |  |
| Lowest Stratum of Probabilities | 82 (28.0%) | 16 (29.6%) | 0.81 |
| Middle Stratum of Probabilities | 64 (32.5%) | 43 (29.9%) | 0.61 |
| Highest Stratum of Probabilities | 53 (59.6%) | 108 (77.7%) | 0.003 |
| Any history of combat exposure |  |  |  |
| Lowest Stratum of Probabilities | 98 (33.4%) | 17 (31.5%) | 0.78 |
| Middle Stratum of Probabilities | 33 (16.8%) | 28 (19.4%) | 0.52 |
| Highest Stratum of Probabilities | 89 (100%) | 139 (100%) | 1.00 |
| Any history of military sexual assault |  |  |  |
| Lowest Stratum of Probabilities | 86 (29.4%) | 20 (37.4%) | 0.26 |
| Middle Stratum of Probabilities | 142 (72.1%) | 106 (73.6%) | 0.75 |
| Highest Stratum of Probabilities | 45 (50.6%) | 47 (32.8%) | 0.01 |
| Any PTSD chart diagnosis, 1994-2006 |  |  |  |
| Lowest Stratum of Probabilities | 42 (14.3%) | 18 (33.3%) | 0.001 |
| Middle Stratum of Probabilities | 197 (100%) | 144 (100%) | 1.00 |
| Highest Stratum of Probabilities | 89 (100%) | 139 (100%) | 1.00 |

PTSD = posttraumatic stress disorder SC- = not service connected for PTSD; SC+ = is service connected for PTSD. Lowest Stratum = probabilities for Time 3 PTSD service connection < 0.38; Middle Stratum = probabilities 0.38 to 0.55; Highest Stratum = probabilities > 0.55.

**Supplemental Figure 1.** Change in the Distribution of Total Disability Ratings by Time, Stratified by Sex.
